# Accessing Properties of Molecular Compounds Involved in Cellular Metabolic Processes with Electron Paramagnetic Resonance, Raman Spectroscopy, and Differential Scanning Calorimetry

**DOI:** 10.3390/molecules28176417

**Published:** 2023-09-03

**Authors:** Eugene B. Postnikov, Michał Wasiak, Mariola Bartoszek, Justyna Polak, Andrey Zyubin, Anastasia I. Lavrova, Mirosław Chora̧żewski

**Affiliations:** 1 Theoretical Physics Department, Kursk State University, Radishcheva St. 33, 305000 Kursk, Russia; 2 Department of Physical Chemistry, University of Lódź, ul. Pomorska 165, 90-236 Lódź, Poland; michal.wasiak@chemia.uni.lodz.pl; 3 Institute of Chemistry, University of Silesia in Katowice, ul. Szkolna 9, 40-006 Katowice, Poland; mariola.bartoszek@us.edu.pl (M.B.); justyna.polak@us.edu.pl (J.P.); 4 Sophya Kovalevskaya North-West Mathematical Research Center, Immanuel Kant Baltic Federal University, Nevskogo St. 14, 236041 Kaliningrad, Russia; azubin@kantiana.ru (A.Z.); anlavrova@kantiana.ru (A.I.L.); 5 Saint-Petersburg State Research Institute of Phthisiopulmonology, Ligovskiy Prospect 2-4, 194064 Saint Petersburg, Russia

**Keywords:** cell wall, *Mucobacterium* sp., antioxidants, EPR, Raman, DSC

## Abstract

In this work, we review some physical methods of macroscopic experiments, which have been recently argued to be promising for the acquisition of valuable characteristics of biomolecular structures and interactions. The methods we focused on are electron paramagnetic resonance spectroscopy, Raman spectroscopy, and differential scanning calorimetry. They were chosen since it can be shown that they are able to provide a mutually complementary picture of the composition of cellular envelopes (with special attention paid to mycobacteria), transitions between their molecular patterning, and the response to biologically active substances (reactive oxygen species and their antagonists—antioxidants—as considered in our case study).

## 1. Introduction

In the last decade, more and more approaches dedicated to the introduction of physicochemical methods for the study of biological systems, from micro- to macrostates (for review, see e.g., [1,2,3,4] ) were developed. In principle, the physical methods themselves for the study of various substances have been under development for a long time; however, their adaptation to biological systems is still the subject of top discussions, since the object under study is unstable, labile and has dynamics.

Among a variety of such studies of different biological systems, we focus on two principal topics: (i) exploring the specificity of the molecular structure and stability of cell membranes as well as the properties of the constituents of the latter, and (ii) characterising the molecules of substances having antioxidant properties and their action on an organism’s tissues due to intermolecular interactions.

Note that both these topics are closely interrelated. The oxidative stress is induced by an elevated chemical reactivity of chemical compounds containing unpaired electrons [5,6,7]. Among them, reactive oxygen species (ROS), which include oxygen and hydroxyl radicals, and superoxide anion are of valuable importance. ROS interact with many cellular compartments including DNA, proteins, and lipids and may result in damages accompanying ageing [8,9], inflammatory processes [10,11], and cancer [12,13]. However, such a process has not only negative but positive effects when it is involved in the antibacterial drugs’ action. The paradigmatic example of such biochemical and biophysical mechanisms is the binding of a first-line antituberculosis drug, isoniazid, to the catalase-peroxidase (KatG) of *Mycobacterium tuberculosis* leading to the inhibition of the biosynthesis of the cell wall constituents [14]. However, the recent spread of drug-resistant strains of this mycobacteria states the complex problems of characterisation of not only the genetic but also the structural and biophysical mechanisms in charge of resistance, especially related to a cell’s envelope, and the need to search for new targets for treatment [15,16].

On the other hand, polyunsaturated fatty acids included in the content of cell membranes are typical molecular targets susceptible to free-radical-attack-induced lipid peroxidation [17,18] that leads, among other effects, to the alteration of membrane fluidity; see, e.g., [19,20,21] for the recent state of the art. The cell has a natural antioxidative protection system, which reduces the effects of free radicals. Among the principal components of this system, there are carotenoids, vitamins, enzymes, and other substances. The antioxidative capacity is related to nutrition, lifestyle, and individual physical condition [22,23]. Therefore, studying the antioxidant action of different substances and their interactions with the cell molecular machinery is considered one of the hot therapeutic topics [24]. Thus, it is promising to study the antioxidant activity not only from the point of view of developing new substances to reduce oxidative stress in normal cells, but also new drugs to suppress the activity of important bacterial enzymes that prevent the appearance of active radicals and the destruction of their cell wall.

The existence of a range of the biophysical and biomedical problems mentioned above induces a demand for developing new approaches based on physical methods aimed at better understanding the respective molecular mechanisms. We would like especially to pay attention to those methods, which utilise “relatively simple” widespread-in-labs equipment of common application rather than a complicated specific one. Three experimental techniques were chosen: electron paramagnetic resonance spectroscopy (EPR), Raman spectroscopy, and differential scanning calorimetry (DSC), which recently gained attention in the biophysical biomolecular research of cell envelopes (in vivo as well as considering in vivo model systems such as liposomes) and their interaction with biologically active substances [25,26,27].

The review is organised as follows. In the first three following sections, we address some specific sides of the application of each of the three considered experimental methods, especially focusing on their less known but prospective applications. For the EPR, it is the characterisation of the antioxidant capacity of substances, which looks like a promising alternative to conventional biochemical and UN–vis spectral methods. For the Raman spectroscopy, we highlight the advantages given by the surface enhanced Raman scattering (SERS), for revealing in detail the molecular features of cells’ envelopes especially responsible for the fast and accurate bacterial strains’ (focusing on mycobacteria species as a valuable public health case) classification giving, among other things, premises for solving the problem of drug resistance. For the DSC, we consider applications to both case examples: mycobacteria and the problem of lipid-centred drug therapy and the antioxidant activity and action.

## 2. Electron Paramagnetic Resonance

The key premise which makes it possible to detect the effect of the electron paramagnetic resonance (EPR) consists of the presence of unpaired electrons in molecules. Although modern development of this technique allows one to construct such conditions for a wide range of systems by the spin-labelling methods, making EPR widely applicable for studying biomolecules and biochemical reactions [26,28,29,30], there are biological systems which are “native” targets for this method. The main types of endogenous paramagnetic centres detectable in the organism in such a way are free-radical-type paramagnetic centres and complexes involving transient metal ions. We mainly focus on addressing recent works which deal with the first kind of species, and especially free radicals and antioxidants at a glance, using EPR spectroscopy. High levels of free radicals, generated in the metabolic processes, can cause cell damage leading to serious diseases such as cancer [12,13], neurodegenerative diseases, diabetes [31], gastric erosions [32], Alzheimer’s disease, arthritis, and ischaemia-reperfusion tissue damage [31,33,34]. Reactive oxygen species ROS (·OH, $O_{2}\cdot$, $H_{2}O_{2}$) are entities that are extremely unstable and react rapidly with other substances in the body, thereby causing damage to cells and tissues [35].

EPR spectroscopy is a valuable tool for studying oxidative stress and antioxidant capacity in cells and biological systems. This technique can directly detect and quantify free radicals, allowing us to assess the levels of oxidative stress in cells and tissues [36]. In addition, EPR spectroscopy can be used to measure the antioxidant capacity of various substances, such as natural compounds, synthetic antioxidants, and foods [37,38,39,40,41,42,43,44,45,46,47,48,49,50].

Moreover, EPR spectroscopy may be used to identify potential pro-oxidants [51,52] and to explore them in therapy, e.g., the photodynamic treatment of various skin diseases [53] including tumours [51,54].

### 2.1. EPR Spectroscopy for Studying Antioxidant Capacity

**Figure 1:**
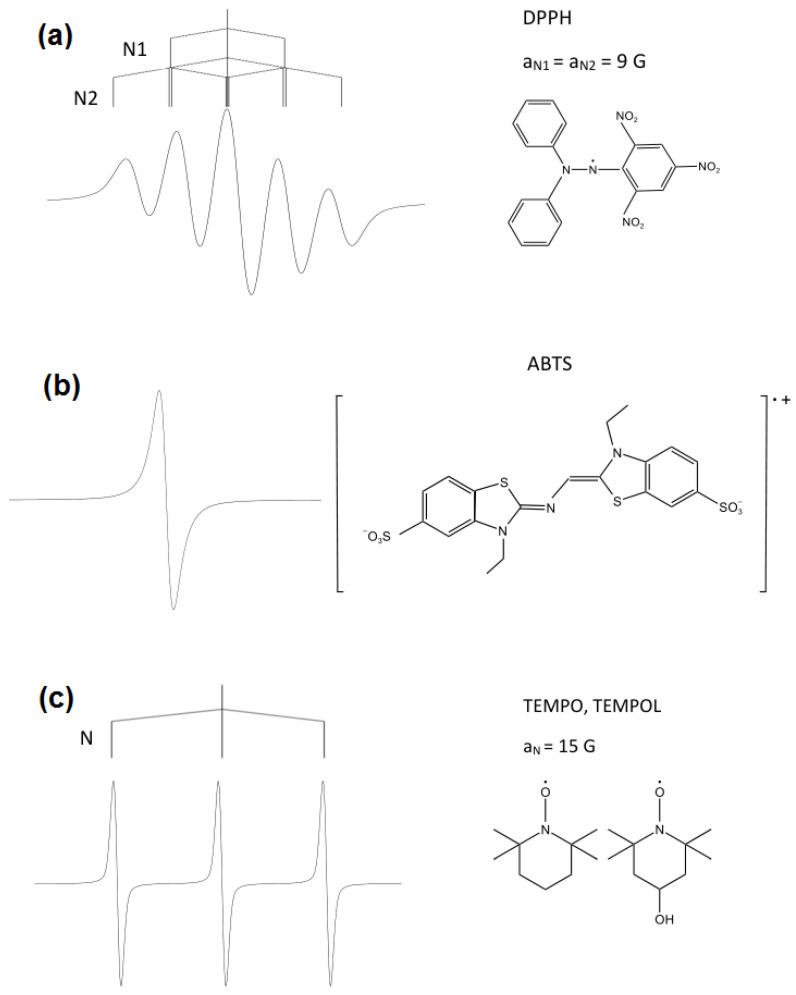
Chemical structures and hyperfine structure in the EPR spectrum of stable radicals commonly used to measure antioxidant capacity: (**a**) DPPH, (**b**) ABTS, (**c**) TEMPO, TEMPOL.

EPR spectroscopy is a technique rarely applied to determine the antioxidant capacity though it seems the most adequate in the case of free radicals. This property originates from the fact that this analytical technique is specifically sensitive to free radicals since it allows for the measurement of transitions of unpaired electrons in a magnetic field. One common method for evaluating antioxidant capacity using EPR spectroscopy is the DPPH (2,2-diphenyl-1-picrylhydrazyl) assay (Figure 1a). This free radical is specially adjusted for testing the antioxidant properties of antioxidants [55] and evaluating the antioxidant activity [56,57]. At the same time, the radical is artificial and cannot be detected in in vivo systems.

**Figure 2:**
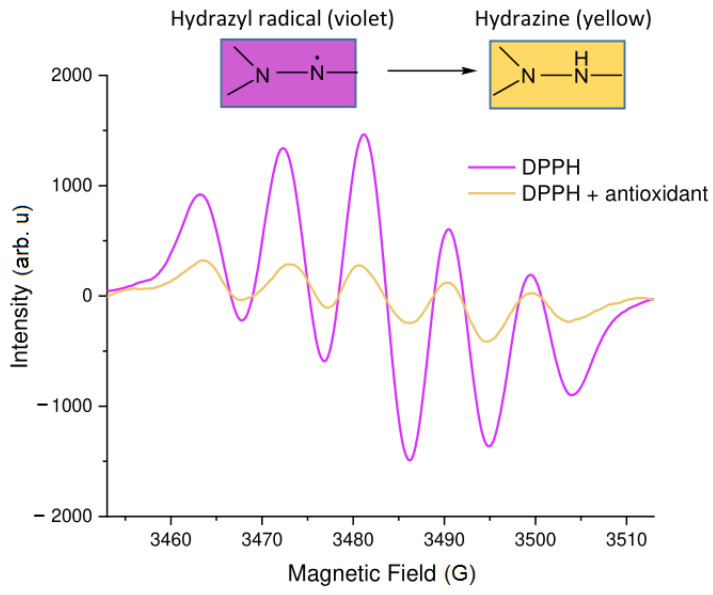
The effect of antioxidants on the EPR spectra of DPPH.

The EPR spectrum of DPPH· in a solvent consists of five lines almost equidistant, with relative intensities of 1:2:3:2:1, which mainly correspond to two equivalent nitrogen nuclei, with the hyperfine coupling constants $a_{N_{1}}=a_{N_{2}}=9\;G$ (Figure 1a), where $a_{N_{1}}$ and $a_{N_{1}}$ are hyperfine coupling constants derived from equivalent nitrogen nuclei. The DPPH method involves adding this radical to an antioxidant sample and measuring the decrease in the EPR signal intensity of the radical as it is scavenged by the antioxidant (Figure 2). The main principle of this assay consists of detecting the reduction of DPPH· to ${\mathrm{DPPH}}_{2}$ when DPPH accepts a hydrogen (H) atom from the scavenger molecule, i.e., the antioxidant. The respective concomitant decrease in absorbance leads to a colour change from purple to yellow. At the same time, the usage of EPR spectroscopy for monitoring the antioxidant capacity is relatively scarce. Among the known studies, one can list investigations of tea leaves [37,38,39], coffee [40], wine [58,59,60,61,62], honey [41], beer [42], tinctures [43], and fruits and vegetables [43,44].

A new method for determining the TEAC (Trolox equivalent antioxidant capacity) based on a new semiempirical mathematical model is a promising alternative to the standard electron paramagnetic resonance (EPR) method. The model was developed by fitting it to 1000 data points of EPR spectroscopy measurements from various food products [48]. The proposed method is fast, accurate, inexpensive, and simple in realization. This approach demonstrated its advantages when applied for the determination of the antioxidant capacity in the cases of alcoholic beverages (beers, wines, tinctures, and strong spirits including whisky, brandy, cognac, vodkas, and liquors) and different types of tea [49,50].

Another method that can be used to evaluate the antioxidant capacity using EPR spectroscopy is the ABTS (2,2’-azino-bis(3-ethylbenzothiazoline-6-sulphonic acid) assay. The concentration of ABTS radical, just like DPPH, decreases due to the action of antioxidant samples [41,63].

${\mathrm{ABTS}}^{\cdot +}$ is a persistent radical that can be readily detected and identified by its characteristic EPR spectrum [64,65]. The ${\mathrm{ABTS}}^{\cdot +}$ radical exhibits a complex spectrum of overlapping lines because of the hyperfine coupling to two sets of equivalent nitrogens and six aromatic hydrogens. The spectrum, centred at g=2.0036±0.0004, is displayed in Figure 1b.

The TEMPOL radical (4-hydroxy-2,2,6,6-tetramethyl-1-piperidinyloxy) is also used to determine the antioxidant capacity using EPR spectroscopy. The EPR spectrum of the TEMPOL free radical in the solvent consists of three lines with intensities of 1:1:1, which correspond to the hyperfine interaction of an unpaired electron with a nitrogen nucleus (Figure 1c) [40]. The antioxidant properties of food products using EPR spectroscopy and ABTS and TEMPOL radicals were determined for wines, tokay [61], honey [41], and coffee [40].

Finally, it is worth noting a possibility to use special biocompatible probes, e.g., aminoxyl radicals (nitroxides) or hydroxylamines, for the purpose of quantifying the number of reactive oxygen species and the organism’s antioxidant defences in vivo. A comprehensive discussion of this specific approach can be found in the review [66].

### 2.2. Biomedical Application of EPR Spectroscopy

In addition to testing the antioxidant capacity in vivo, EPR spectroscopy has found applications in various branches of medicine. Paramagnetic metal complexes and free radical compounds are involved in the functioning of various organ- and tissue-related biochemical systems. Since they have specific EPR spectra, this allows one to carry out noninvasive EPR imaging.

Applications include the detection and imaging of endogenous free radicals such as TEMPO or PROXYL. The experiments are carried out in the X-band (ca. 9.5 GHz) and in the L-band (ca. 1 GHz). EPR imaging is used in studies of small animals such as mice and rats [67,68]. The nontoxic character of specially chosen markers allows the tracing of the EPR signal during sufficiently long time intervals and the analysis of various kinds of ROS-related metabolic processes in their continuity. The work [69] provides multiple example of such investigations, among which the detection of cytochrome P450 overexpression and the consequences of the dietary supplement β-carotene. The works [33,70,71,72,73] report both the construction of whole-body clinical EPR systems and adapted resonators, which provides the possibility to be applied in human-body-related EPR studies. In such research, EPR imaging can determine tissue parameters including oxygen partial pressure, the redox microenvironment, reactive oxygen species, and free radical melanin as a potential diagnostic parameter for skin disease. A wide variety of suitable paramagnetic probes applicable for EPR imaging exist: they have simple EPR spectra, are well tolerated, and have a half-life time that is longer than the imaging time [68,74].

Moreover, the EPR method has been used in dermatology [75,76] including the study of skin cell (e.g., keratinocytes) damage by technical nanoparticle pollutants [77]. Yellow, orange, and red organic pigments are present in a wide variety of natural objects, e.g., plants and animals, and are known as substances characterised by a high-level antioxidant activity by singlet oxygen and peroxyl radical quenching [78,79,80]. This makes them attractive as biologically active food components, additives to photoprotective cosmetics, etc., [81,82,83,84]. The chemical structure of carotenoids and their radical-related reaction allows one to quite clearly access the involved unpaired electrons by the EPR, which ensures this method is accurate and informative to the related investigations [85,86]. Concerning cellular membranes and their compartments, the review [87] summarises earlier works on the features revealed by the EPR methods, among them, one can mention the following: polar carotenoids can either increase or decrease the membrane fluidity depending on its phase state, ordered phase or liquid crystalline, respectively. Recently, this interpretation of the EPR-based data received a detailed confirmation of microscopic mechanisms by all-atom MD simulations [88].

**Figure 3:**
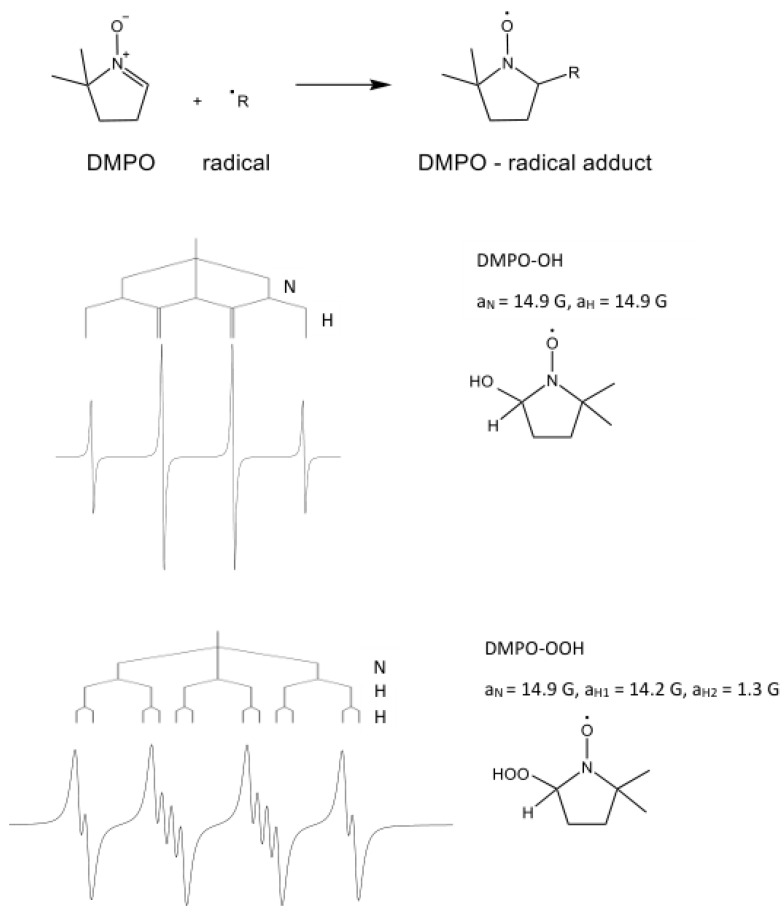
DMPO spin adduct formation reaction and simulated EPR spectra of DMPO radical adducts.

The direct detection of reactive oxygen species (ROS) playing a critical role in many biological processes, such as OH·, $O_{2}^{-}\cdot$, ROO·, RO·, ${\mathrm{CH}}_{3}O\cdot$, and ${\mathrm{CH}}_{3}\cdot$, is not possible by EPR spectroscopy, because the ROS are short-lived and do not accumulate. The study of these radicals is possible using spin-trapping techniques [89]. This method consists in the ROS being “trapped” or stabilized by the addition of a radical spin trap, which forms a stable adduct with the radical, which lives long enough for the spin trap adduct to be measured by EPR. Among the widely used spin traps applied for the study of oxygen-centred free radicals in biochemical and biological systems is 5,5-dimethyl-1-pyrroline N-oxide (DMPO). It allows the exploration of the formation of superoxide ($O_{2}^{-}\cdot$) and hydroxyl radicals (·OH) as well as peroxyl radicals [89]. Figure 3 demonstrates that the spectra of spin adducts of selected DMPO radicals are different; the number of lines in the EPR spectrum and coupling constants changes depending on the trapped radical.

The spin-trapping method is used to study the biochemical reactions themselves (e.g., the process of photosynthesis or the respiratory chain pathway) and the products formed during the metabolism of drugs and pathogenic processes [90,91]. For human studies, one can use spin-trap additions to blood, plasma, urine, tissue biopsies. In fact, it is the only method that can be currently employed [92,93,94], but it encounters significant difficulties in the interpretation. These complications originate from the lifetime of any radicals being shorter than even the fastest sampling procedure. Thus, an interpretation is required for detected signals which arise from secondary processes [92].

Moreover, using spin labels, EPR spectroscopy has been successfully applied to probe the molecular interactions between proteins and polyphenols at the molecular level [36]. Spin labels are paramagnetic centres attached to selected elements of biopolymers. The markers can bind to the tested system by forming covalent bonds, forming complexes, or by physicochemical interactions. Spin labels contain two centres: a reactive centre enabling the formation of a covalent bond, and a paramagnetic centre (usually a nitroxide radical). TEMPO and TEMPOL are the most commonly used spin markers [95].

Additionally, as a spin trap, DEPMPO can be applied [96,97]. This compound is a phosphorylated derivative of DMPO and can be considered as its kind of improvement. Its principal advantage is a longer lifetime, especially for a superoxide adduct. In addition, DEPMPO is more stable than the DMPO spin trap, especially in vivo, and appears to be a better spin trap in functioning biological systems. These effects motivated its usage in intact animal models, see, e.g., the modern review [98].

Using site-directed spin-labelling electron paramagnetic resonance (SDSL-EPR) is possible. The SDSL-EPR technique involves introducing a paramagnetic probe (usually a nitroxide spin label) at a specific site in the biomolecule of interest, which can then be detected by EPR spectroscopy. By monitoring the EPR spectrum of spin labels, information about the local environment and mobility of the labelled site can be obtained and can be used to infer structural and dynamic changes in the biomolecule [99].

## 3. Some Modern Prospective Applications of Raman Spectroscopy

Raman spectroscopy is a widely used research method since its application makes it possible to obtain an accurate spectral “fingerprint” of the molecular structure of molecules. A high-frequency Raman spectrum represents a set of frequency shift peaks corresponding to certain oscillations of molecular bonds and, therefore, every such peak coincides with an element of a molecular structure, and the peaks’ modifications reveal structural changes due to chemical reactions of other influences.

Currently, more than 20 different Raman spectroscopy techniques are known [100]. These include spontaneous Raman scattering, which is the most common, and resonant Raman scattering, operating with an excitation near the molecular absorption band, which allows signal amplification. Also of note is the Fourier transform Raman used to make the Raman signal clearer and improve the signal-to-noise ratio, and confocal Raman spectroscopy for mapping the surface (or subsurface) layer, often used in biophysical studies of cells, bacteria, and tissues. Finally, Raman spectroscopy with surface enhancement, based on the plasmon-mediated amplification of electric fields by the metal of nanostructures, should be noted, which leads to a gigantic increase in sensitivity.

For this reason, Raman spectroscopy has recently attracted growing attention in biochemical and biomedical research including cell biology and microbiology [27,101,102,103,104,105]. The prospective areas of application in this field are sufficiently wide, see, e.g., the reviews cited above, but below, we restrict ourselves to those which are coordinated by their objects of investigation with other methods considered in our work.

### 3.1. Raman Spectroscopy for Mycobacterial Studies

To date, a wide range of bacterial cells have been investigated, and techniques for the differentiation of various bacterial cultures have been successfully implemented [106]. Moreover, the origin of the possibility to use the SERS method for this goal was discussed from the biochemical point of view [107] and from that of distinguishing between microbiological and physical factors by determining the interactions between biomolecular constituents and the signal-enhancing substrate [108]. Within the framework of the current review, we restrict what follows to the growing interest in the application of Raman spectroscopy based on the giant scattering to studies of mycobacterial cultures and individual cells. This interest originates from the biomedical demand for developing fast and accurate tools for distinguishing between tuberculosis bacterial species and detecting interspecies and interstrain differences.

In particular, the authors of the work [109] proposed an approach to the differentiation of tuberculous and nontuberculous mycobacteria using a combined lab-on-chip Raman technique, which allowed them to obtain more than 2100 spectra per hour. The resulting massive quantity of data led to them to build an identification tree based on the chemometric model formed on the basis of the database of spectral features. Stökel et al. [110] investigated two fast-growing species of mycobacteria, *Mycobacterium aurum* and *Mycobacterium smegmatis*, which can serve as a proxy for biomedical studies during the growth of cultures, and revealed the possibility of distinguishing between them due to the different ways of uncovering the molecular fingerprints of the lipid composition at the late stage of growth. This fact confirmed the importance of mycolic acid components contained in the cell’s outer membrane as was demonstrated in the work [111] using lipophilic extracts from different mycobacterial cultures. Later, in a continuation of this avenue of research [112], 26 *Mycobacterium* species (39 strains) divided into nontuberculosis mycobacteria (NTM) (24 strains) and bacteria of the *Mycobacterium tuberculosis* complex (15 strains) were investigated by operating on the respective colonies and a spectral library was created. Using chemometric methods of spectra differentiation, the accuracy of species determination at the NTM-/MTB-species level varying from 88% to 99% was achieved. As an example of such fast practical diagnostic applications, one can list the work [113], where it was shown that Raman spectroscopy provided an opportunity to detect *M. tuberculosis* in cerebrospinal fluid samples and diagnose tuberculous meningitis in such a way.

The authors of the work [114] investigated a cell wall component of *M. tuberculosis*, mannose-capped lipoarabinomannan, which is a significant component of the mycobacterial cell wall and, simultaneously, a major virulence factor in the infectious pathology of tuberculosis. Thus, it has been considered as a possible biomarker, which can be determined in a patient’s serum using the SERS-based immunoassay. A potential of such an approach for diagnostic purposes has been demonstrated despite revealing some uncertainties when operating with real clinical data originating from the variability of treatment considerations and specificity of a person’s health conditions. The subsequent research in [115] resolved some of these issues by formulating a more specific protocol for the pretreatment of the samples.

In the work [116], the authors provided a sequential procedure for the determination of biomarkers in mycobacterial cultures using SERS with the Ag-covered silicon-nanopillar substrate. Mycolic acids were considered as the biomarker. A library of Raman spectra of its principal form (e.g., alpha-, methoxy- and keto-mycolic acids) was formed and then used as the reference for results obtained for extracts of MbT. When addressing compounds of the cell wall, an original method of the signal enhancement based on the usage of silver nanoparticles was developed in the work [117], where the silver mirror reaction created silver nanostructures enhancing Raman signals directly on the surface of the bacterium. This approach allowed the identification of pathogens *M. bovis BCG*, *M. tuberculosis*, *Staphylococcus aureus*, *S. epidermis*, *Bacillus cereus*, and *Escherichia coli*. Apart from the cell wall constituents, the DNA was also considered as a biomarker for the rapid detection of *M. tuberculosis* by means of surface-enhanced Raman spectroscopy, see, e.g., the work [116], where a sensor formed by gold nanorods embedded in graphene 3D matrices was implemented to identify target DNA fragments.

Another recently developing direction of applications of Raman spectroscopy is studying the action of antimycobacterial drugs and drug resistivity. For the first item, spectroscopic methods are applied to characterise the acting molecules and the biochemical reactions of their binding. To list some of the representative works, it worth noting the study by Rawat et al. [118], where quantum mechanical modelling of the spectral and energetic characteristics of pyrrole-isonicotinyl hydrazone was performed in comparison with results of different spectral methods including FT-Raman to reveal the principal antimycobacterial pharmacophore group of this substance. The standard reference strain *Mycobacterium tuberculosis* H37Rv was used for testing. In the work by Thomas et al. [119], a detailed theoretical study of pyrazole derivatives by the method of density functional theory (DFT) was carried out in order to identify the features of molecular dynamics and molecular docking to the binding centres of mycobacterial proteins. The authors carried out a modelling of vibrational modes and identified characteristic maxima revealing the binding of pyrazole derivatives and mycobacteria. Robert et al. [120] used different methods, including Fourier transform Raman spectroscopy (FT-Raman), to get a full characterisation of the antibacterial agent PYCA that was required to discuss theoretically its structure and binding features accompanied with the test of its antibacterial action on *M. stegmatis*. In summary, the growing collection of such spectral data containing fingerprints of different pharmacophore groups opens perspectives for a future targeted synthesis by application of AI-based tools [121].

On the other hand, the active modern topic is the usage of Raman spectroscopy for the fast detection of drug resistivity. This interest is based on the assumptions that different statuses of resistivity depend on the composition of a cell wall and the detection of structural and biochemical changes, which can be reflected in the spectral properties faster than they can be detected by conventional methods of a culture’s growth [122]. In addition, such specificity can be studied even for an individual bacterium. Although there are known attempts at the usage of SERS for different bacterial species, see, e.g., [123,124], the case of *M. tuberculosis* is more complicated and represents a recently emerging area, which belongs to the so-called “Big 5” challenges of fighting diseases induced by drug-resistant bacteria [125].

In particular, the work [126] discusses the application of machine learning methods for the classification of spectra obtained within the framework of SERS fingerprinting (note also that the idea of using machine learning tools such as deep learning was implemented earlier for other types of bacteria as well [127]). It has been shown that it is possible not only to successfully distinguish between sputum samples with or without MtB strains (fivefold cross-validation accuracy = 94.32%) but also to differentiate strains isolated from pulmonary and extrapulmonary MtB samples (fivefold cross-validation accuracy = 99.86%) and most importantly, to separate MtB strains with different drug-resistant profiles (fivefold cross-validation accuracy = 99.59%). The work [128] focused on rifampin insensitivity associated with tuberculosis resistance to rifampicin, one of the most common antituberculosis drugs. The authors proposed to combine SERS with the polymerase chain reaction (PCR), achieving a fast detection of the presence of wild-type and mutated genes responsible for resistance (96% sensitivity, 95.6% specificity, and 95% accuracy). On the single-cell level, the work [129] announced the possibility to reduce with the usage of Raman spectroscopy the time required to detect the resistance to the major first-line antimicrobials rifampicin and isoniazid from 7 days to 12 h. The work by Ueno et al. [130] successfully demonstrated (using *M. smegmatis* as an example) that Raman spectroscopy also allows the tracing of the time dynamics of a growing and dividing mycobacterial cell and the measurement of the metabolic activity of rifampicin-tolerant *M. smegmatis* in time dynamics.

**Figure 4:**
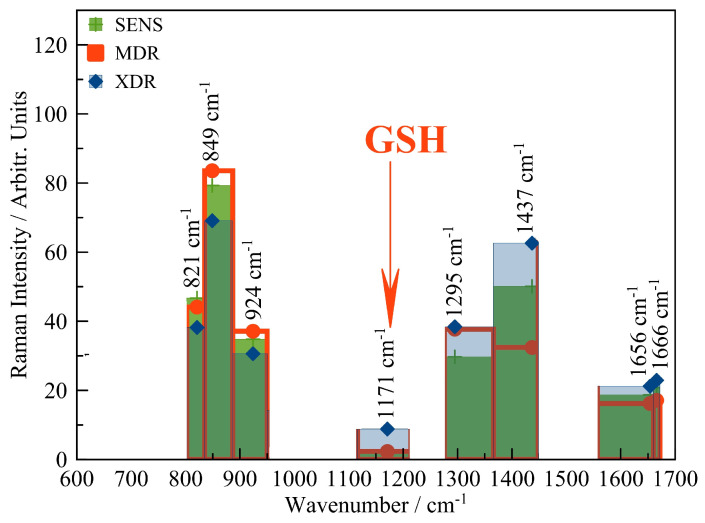
Several representative spectral lines recorded by the SERS method from samples of pulmonary strains of *M. tubeculosis* with different drug resistance statuses (see Ref. [131] for the full characterisation of the data used). One of the specific lines hypothesised as belonging to the spectrum of glutathione is highlighted with the arrow and label “GSH”.

Apart from the purely data-based research, which utilises big data and various machine learning approaches for distinguishing between drug-susceptible and drug-resistant strains of *M. tuberculosis*, there is also a series of work focused on the revealing specific molecular features of the cell wall, which explain the difference in the investigated Raman spectra underlying the classification process. They started from the work [131], where six spectra of clinical samples of sensitive, multidrug-resistant, and extensively drug-resistant MbT (Beijing family) isolated from respiratory and surgical material were obtained and analysed. It was concluded that the different statuses of drug resistivity could exhibit themselves in a varying amplitude of certain bands belonging to both cell walls and DNA, see Figure 4. The former was investigated in more detail in subsequent work [132]. The results of this study argue that the desired difference can be detected in the high wave number region corresponding to the vibrational stretching of lipids and proteins. This supports the importance of characterising the specificity of the lipid-based constituents of the cell envelope by different methods, including those which are considered in the present review. As another promising target, glutathione was revealed [133] in the region of moderate wave numbers (1000–1500 ${\mathrm{cm}}^{-1}$). Figure 4 highlights one of the spectral lines belonging to the glutathione spectrum, which is a rather stable representative of this molecule under various conformations and external conditions [134,135] and, simultaneously, clearly exhibit the regular amplitude growth when *M. tuberculosis* has extra drug resistivity. From the biochemical point of view, it is well known as one of the key natural cellular antioxidants [136] also actively involved in the process of tuberculosis infection [137] and curing [138]. Thus, this fact is an argument in favour of studying the complex joint molecular processes in cells’ envelopes and the molecular biochemistry of antioxidative compounds.

Finally, it should be pointed out that in addition to diagnostic purposes, the exploration of Raman spectra provides outlooks for developing microscopic models of drug action and resistance as described in the work [139] tracing the time evolution of the Raman spectra obtained from a culture of *M. smegmatis*, a model organism for M. tuberculosis growing either under standard conditions or under the influence of isoniazid in a microfluidic setup.

### 3.2. Raman Spectroscopy for Studying Antioxidants and Their Biochemical Action

Since Raman spectroscopy is a method adjusted for detecting molecular fingerprints of DNA, proteins, lipids, and other cell constituents, which can vary when cells are influenced by different factors, this method of investigation found its application in studying both biochemical processes induced by oxygen reactive species and compounds acting as antioxidants. An additional feature, which differs from the Raman effect based method, is the possibility to choose well-localised positions, i.e., to highlight spatial features of such processes. This fact resulted in a wide usage of Raman spectroscopy for the exploration of the related biochemical processes in living tissues, such as skin and retina tissues, when the excitation ray is focused on a particular spot of the tissue, and the selective processes of oxidative stress and antioxidative protection were revealed, including the detailed study of antioxidants’ penetration; see the works [140,141,142,143], which established this research direction.

Among the recent studies, one can highlight Raman microspectroscopy, i.e., recording signals acquired from particular places of one cell [144]. This allows one to obtain a quantitative spatiotemporal resolution of the oxidative damage and antioxidant protection against it in a precisely localised place of the cell. In addition, this information allows a discussion of cytokine/chemokines-involved biochemical mechanisms in the respective inflammatory response using methods of statistics and machine learning. Another example of the Raman supported discussion of biochemical processes is given by the work [145]. Its authors addressed the process of lipids’ oxidation and protective action of antioxidants in the skin’s surface layer and analysed responses of different molecular bonds to increasing doses of photoradiation. This resulted in the emergence of metabolite “fingerprints” of damaging oxidative stress, as well as those corresponding to the interaction of peroxyl radicals with squalene, cholesterol, vitamin E, and palmitoleoyl palmitate. The dependence indicated by a number of such metabolic molecular fingerprints (including the penetration depth) was considered in the work [146]. A specially developed method of matrix factorization allowed the assessment of the concentration profiles of the damaged molecular skin constituents and protective antioxidant species. Note that the approach which refers to a number of Raman spectral components was also recently demonstrated as a prospective approach for characterising the drug resistivity of *M. tuberculosis* [147], one of the mechanisms of which is related to the oxidative damage of the cell wall. These emerging studies argue in favour of the consideration of not only Raman spectral components themselves but also the complexity of a spectrum’s composition. A detailed profiling of the penetration of two antioxidants, retinyl acetate and alpha-tocopheryl acetate, which are vitamin derivatives, is presented in the work [148], utilising the ability to focus laser rays in different depths of an investigated medium, so-called confocal Raman spectroscopy. The distribution and aggregation of carotenoids in lipid bilayer lamellas of the stratum corneum (a protective layer of the skin) was constructed in the work [149]. This opens perspectives for a detailed molecular modelling of the respective biomolecular processes, even prospectively, with an application of methods of molecular dynamics or density functional theory. It is worth noting that the required molecular structure of several carotenoids was recently discussed regarding their antioxidant properties [150,151] by applying Raman spectroscopy as well.

A special interest is also on the applications of surface-enhanced Raman scattering (SERS), which is, as described above, a powerful tool for exploring biophysical issues related to the molecular composition of bacterial cells. Within the context of antioxidant studies, this method is useful too, primarily for the characterisation of molecular features of antioxidants. In particular, the authors of the work [152] performed SERS measurements for ferulic acid, p-coumaric acid, caffeic acid, and sinapic acid. They prepared and characterised SERS-active substrates, carried out a PCA and, as a result, developed a highly sensitive SERS-based method for phenolic antioxidant detection. Another common food antioxidant—butylated hydroxyanisole (BHA)—can also be detected using SERS [153] at the qualitative and semiquantitative levels. This enhancement of the signal, corresponding to the $480\;{\mathrm{cm}}^{-1}$ maximum, results in a minimum detection limit of 10Lg/mL for BHA. A SERS-based method for the multiphase detection of antioxidants was further developed by Q. Tu. et al. [154]: a gold nanoparticles-coated fibre was directly inserted into a multiphase system with different model antioxidants. The aqueous, intermediate, and organic phases of a multiphase system were explored for the cases of the addition of ascorbic acid, ascorbyl palmitate, and α-tocopherol. In general, gold and silver nanoparticles (e.g., rods, stars, etc.) gain bursting attention as excellent SERS substrates. A high level of signal enhancement was demonstrated in the study of a variety of typical representative antioxidants by applying an array of such gold nanoelements in the work [155]. A silver nanotripod substrate demonstrated its performance for ultrasensitive measurements of lipid-soluble antioxidants as reported in the paper [156].

## 4. Differential Scanning Calorimetry

The method of differential scanning calorimetry (DSC) finds its application in characterising properties of biomolecules which are microscopically related to their spatial configuration and energetic stability. These features can significantly affect biochemical interactions in general. The DSC method allows one to find the enthalpies of the respective phase properties via a relatively simple induction of transitions by heating and measuring the respective enthalpy.

**Figure 5:**
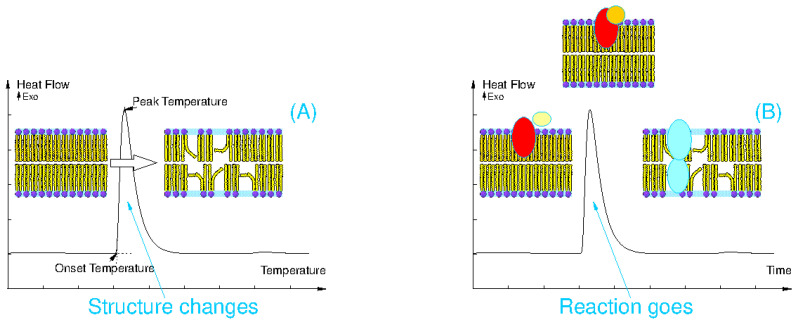
Examples of typical DSC curves, which reflect (**A**) a thermal response to a structural change exemplified as emerging membrane fluidity and (**B**) time course of the isothermal DSC curve emerging due to a biochemical binding of two compounds.

Two principal approaches to the method DSC, which reflects specific information on the molecular composition, structure changes, and chemical and biochemical reaction, are the conventional scanning, which records the response to the change in temperature (Figure 5A) and the isothermal DSC, which keeps a sample at a prescribed temperature and indicates heat flows emerging due to a running chemical reaction (Figure 5B).

Thus, within the principal focuses of this review, we address two particular directions: (i) the DSC-based exploration of biomolecules contained in the cell wall and interaction with its principal constituents, and (ii) the antioxidant activity of different substances including their involvement in physiological processes.

### 4.1. DSC of Molecules of a Cell Wall and Interaction with Them

A cell wall is not a simple envelope bounding its internal content but is actively included in more complex processes of proliferation, division, and interaction with the surrounding environment. As for the latter feature, special attention is warranted to the problem of antibiotic drug resistance of bacteria, which induces diseases heavily burdening public health [157,158,159]. From this variety, we focus on tuberculosis, which is still one of the leading causes of death in the world [160]. It is known [161,162] that the structure of the composition of the mycobacterial cell wall is a necessary (although not unique) factor in charge of resistance to antibiotics’ action. Simultaneously, it should be pointed out that not only *M. tuberculosis* but also other bacteria belonging to the genus *Mycobacterium* attracts attention, being considered as less pathogenic and, respectively, more easily usable model organisms.

One of the cell wall’s features, which can be acquired using the DSC method, is an organization of the constituent lipids, especially mycolic acids and peptidoglycan complexes. Although the static tiling can be accessed by X-ray diffraction studies, the dynamic properties expressed as fluidity can be captured by using the thermal response recorded by dynamic scanning. For *M. chelonae*, is has been shown in the work [163]. The proposed hypothesis that the fluidity of the mycobacterial wall (and, respectively, the permeability of mycobacteria) is significantly determined by the presence of mycolic acid was confirmed by studies described in the work [164] with the DSC for eight mycobacterial species including *M. tuberculosis* strain H37Rv. It should be pointed out that this conclusion was achieved by a thermal analysis without the involvement of more complicated techniques of molecular biophysics but by thermally exploring bacterial samples and reference mycolic constituents. Such a physical approach to the study of biomolecular arrangement was also demonstrated in the work [165], where the authors operated with the Langmuir monolayer formed by mycolic acid extracted from *M. tuberculosis*.

The most recent approaches which demonstrate the utility of the DSC method within this context address the development of new, efficient antituberculosis agents, and the ways of their delivery and action in the framework of the so-called lipid-centric therapy [166,167]. In particular, the authors of the work [168] argued that the mycolic lipid component trehalose 6,6${}^{\prime }$ dimycolate, abundant in MtB’s cell wall, has immunostimulatory activity and forms a basis for antituberculosis vaccine development. Its compatibility with a cubosome lipid nanocarrier used for encapsulating was investigated by the DSC scan analysis of thermograms for the components and their complex. Another variant of the lipid-based encapsulation of antibiotics (including the “classic” first-line drug isoniazid) was proposed in the work [169]; the DSC provided an opportunity to characterise the optimised compatibility of drug and lipids in the composition. The specific form of drug delivery in the form of co-spray-dried powders comprised of the different compositions of capreomycin (a semisynthetic antibiotic) and antimicrobial peptides (i.e., biomolecules) for pulmonary delivery was characterised with DSC in the work [170].

### 4.2. DSC and Studying Antioxidant Action and Activity

In the research on the compounds which exhibit antioxidant properties, differential scanning calorimetry has also found its place as a useful tool. It is mostly used in two ways. The first is to investigate the behaviour of the sample during heating, which allows one to address the structural, conformational, and energetic properties of molecules. The second is to study the heat flow in the isothermal condition during the purging of the sample with oxygen, exploring in such a way the basic chemical and biochemical processes of interest. Obtained from those measurements, the oxidation induction period and oxidation initial temperature can also be modelled using the “hard” (nonlinear regression) and “soft” (projection on latent structures) approaches [171].

It is worth noting that one of the target processes of interest is the interaction of an antioxidant with the cell membrane or its constitutive components; it continues the line of reasoning considered above. In particular, ruscogenin, a natural steroidal saponin has been studied by I. Sahin et al. [172].

The prevention of a lipid’s peroxidation by saponins is based on their amphiphilic nature, which leads to the formation of aggregates interacting with membrane components. Ruscogenin’s interaction with multilamellar vesicles (MLVs) formed by dipalmitoyl phosphatidylcholine (DPPC) and anionic dipalmitoyl phosphatidylglycerol (DPPG) causes considerable alterations in the phase transition profile, as revealed by the DSC analysis. The analysis of this profile’s features allows one to characterise the order, dynamics and hydration state of the head groups and glycerol backbone of DPPC and DPPG. This recent work continued a stream of research devoted to the interaction of various antioxidants with this type of model membranes. The authors list, e.g., the works considering the interactions of some flavonoids, including anthocyanin, with DPPC and DPPC/cholesterol carried out by D. Bonarska-Kujawa et al. [173], and paclitaxel (an antineoplastic drug) with DPPC-based lipid bilayer vesicles (liposomes) investigated by L. Zhao et al. [174]. From such studies, information about the stability of the vesicles, which can be used as a model for cell membranes, and the influence of the additives can be obtained. For example, the results of the latter study indicate that the absence of paclitaxel results in two thermal transitions observed for the DPPC liposomes. However, even a small amount of paclitaxel can eliminate the pretransition of DPPC liposomes. Another strong perturbing effect on the DPPC/paclitaxel mixed bilayer systems is caused by the incorporation of cholesterol. Thus, the DSC analysis shows that paclitaxel, which can be considered as an example of antineoplastic drugs, acts as an agent leading to a looser and more flexible bilayer structure due to a reduction in the cooperativity of the bilayer expressed physically as a phase transition. Simultaneously, the tertiary system, which also includes the cholesterol component, is even more stable.

Another target for the DSC studies is the stability of antioxidant compounds either by themselves or in combination with by-side additives used in the food industry, cosmetics, and similar ways to deliver antioxidants to human organisms. For example, morin, a natural polyphenol, has a higher antioxidant capacity, but after complexation with other food ingredients, the emerged covalent and noncovalent interactions could affect its activity. Natural starch is formed up of linear amylose and highly branched amylopectin. Complexes of amylase and morin (A-Mo) were studied by Y. Ma at al. [175]. They observed two peaks in the DSC curve of the A-Mo complex. This shape drastically contradicted the case of amylose and indicated the better stability of the A-Mo complex. The evaluated analysis argued that the first step transition corresponded to the disintegration of free morin on amylose. The respective interaction between them was rather weak. Only the further growth of the temperature resulted in the collapse of the A-Mo complex’s structure. The thermal behaviour of the UV-A and UV-B filters butyl methoxydibenzoylmethane and ethylhexyl methoxycinnamate with the addition of the antioxidants ferulic acid and resveratrol was studied by T.B. Freire et al. [176]. They concluded that photoprotective and antioxidant efficacy and safety could be compromised. In addition, a possibility of making predictions on the stability profile, as well as an analysis of the long-term interactions of cosmetic ingredients and resulting formulations, was demonstrated.

The thermal-oxidative decomposition of natural products has been studied by many authors. Early studies mainly addressed the demands of the food industry. The oxidation temperature, as well as the induction time of the palm oil stabilized by various antioxidants studied by T.A. Pereira and N.P. Das [177], lead to the claim that palm oil’s resistivity to oxidation did not depend on the degree of saturation, and it exhibited a better oxidative stability than, e.g., olive oil, soybean, and sunflower oil. An additional attention has been paid to the DSC method as an approach for “fingerprinting” oils and fats. B. Kowalski [178] studied edible vegetable oils and lard and three different antioxidants to find the best additive based on the analysis of the onset oxidation temperature and calculated activation energies. Three different types of edible oils were also investigated by C.P. Tan and Y.B. Che Man [179]. The authors analysed the cooling thermograms, especially crystallization peaks and the influence of the long-term heating of the samples at 180 ${}^{\circ }$C. They compared the results with those from a standard chemical analysis and suggested that the DSC method could be an efficient method of monitoring the extent of lipid oxidation. Another eleven vegetable oils were studied by J. Velasco et al. [180] who explored and discussed the oxidative stability, among other approaches, by the DSC method.

Other examples related to this stage of development on the DSC’s usage to assess the oxidative deterioration of vegetable oil products can be found in the review paper [181], which also discusses biochemical kinetics of oxidation as related to the data registered with the differential scanning calorimetry.

Over the last few years, interest in the DSC as a method for accessing antioxidant properties has been actively revitalised by focusing on antioxidants of natural origin, especially those containing fatty acids. Such antioxidants are revealed as constituents of the chemical composition found in many aromatic, spicy, medicinal and other plants. Seventy methanolic extracts from fifty-seven Greek plant species were investigated by E. Kalpoutzakis et al. [182] using the DSC analysis, focusing on their thermal-oxidative decomposition, i.e., the purge gas foaming the reaction atmosphere was oxygen, and an exothermic peak was observed related to the auto-oxidation process of the samples. The statistical analysis of the respective oxidative stability of the extracts supported the build hierarchy of their desired properties. A similar study of the oil extracted from rose hip seeds, considered as a substantial source of unsaturated fatty acids, has been conducted by M. Grajzer et al. [183]. Among these substances, the most abundant were linoleic (35.9–54.8%), a-linolenic (16.6–26.5%), and oleic (14.7–22.1%) acids. All of them had a relatively high protection against oxidative stress. In that study, the oxidative induction time (OIT) and the end time of the induction period were determined by the DSC analysis. It was carried out under isothermal and non-isothermal conditions. In the former case, the induction period onset in oil purged with oxygen or air was detected as a function of time. In the latter, the instant temperature dependence was recorded. The oil’s better oxidative stability and longer shelf life corresponded to the higher OIT values. The antioxidant properties of carnosic acid, present in rosemary, and tertiary butylhydroquinone (TBHQ), a synthetic food grade antioxidant, against the oxidation of oleic acid were studied by J. Wei et al. [184]. The DSC measurements allowed for the determination of the apparent activation energies of the reaction, i.e., to access the change in the reaction rate of oleic acid. Its specificity revealed in the cited work led to the prolongations of the oxidation induction period due to the outcompetion of tertiary butylhydroquinone.

## 5. Combining Different Methods

It should be noted that each of the three physical methods reviewed above has its own strong points highlighting definite biophysical and biochemical molecular mechanisms. Respectively, their combination allows for a more multifaceted process, acquiring and highlighting the details of processes.

### 5.1. Combining EPR and Raman Spectroscopy Methods and Data

As the first example, we note that the spectral data obtained via Raman spectroscopy and electron paramagnetic spectroscopy are sensitive to the change in molecular structure which originates from biochemical reactions involved, e.g., in metabolic processes. Hence, it is not surprising that several studies were devoted to the respective analysis aimed at comparatively revealing the strong points of each approach, their best area of application, and their usage for determining the parameters of kinetic models describing such interactions.

**Figure 6:**
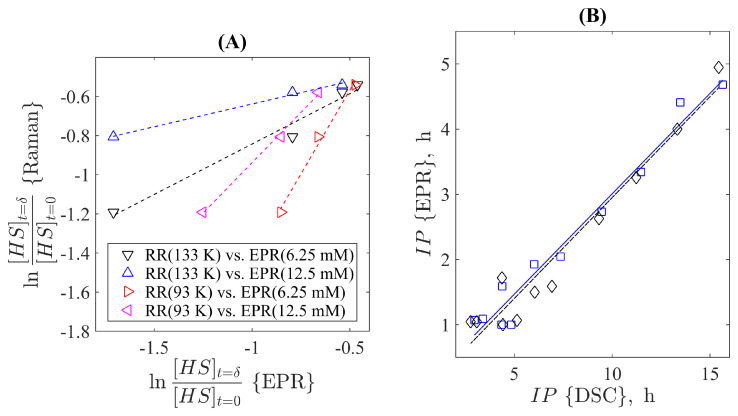
(**A**) An illustration of the linear correlations between the kinetic constant defining the quasi-first-order model for azide binding to myoglobin as determined by resonance Raman and electron paramagnetic resonance spectroscopies under different conditions (the temperature of frozen samples and azide’s concentration). The digitized and reprocessed data reported in the work [185] were used for this plot. (**B**) An illustration of the linear correlation between the induction period of oxidation in different vegetable oils determined by the EPR and DSC methods. The data of two repetitive experiments (blue squares, solid line; black diamonds, dashed line) reported in the work [180] were processed and used for this representation.

The work [185] explored this compliance by directly exploring the time course of the quasi-first-order reaction of the binding between ferric myoglobin and azide (${\mathrm{NaN}}_{3}$). The respective kinetic binding rate was determined by the spectral testing of a series of samples with some time steps after the reaction’s beginning. These samples were fast-frozen (quenched) and the respective resonance Raman (RR) and EPR spectra were determined simultaneously. It was revealed that the magnitude changes of specific spectral lines during the time course exponentially (linearly in the semi-logarithmic coordinates) allowed for the determination of the respective kinetic constant by both methods. They fell in the qualitatively same range of variation although it was revealed that the RR method was more stable, as it was less affected by the concentration of azide influencing the molecular packing ratio during the fast freezing. The conclusion on the advantage of Raman spectroscopy in studying the reaction dynamics of proteins resulted in the more active development of this approach for time-resolving investigations of biomolecular processes [186]. At the same time, it is also possible to demonstrate the compliance between the two methods more illustratively. For this goal, we digitized the data reported in the work [185] for the same reaction time moments and replotted them as a correlation plot directly, see Figure 6A. One can see that the markers already follow the dashed straight lines indicating the linear correlation between them. In addition, these plots visually illustrate the mentioned dependence of the EPR-based data on the concentration of azide and the temperature of quenching reflected as the change in slope. At the same time, the linearity of the dependence is kept for all combinations providing a principal opportunity for reciprocal mapping of the data obtained by these two methods adjusting them to the actual thermodynamic conditions of an experiment.

However, even more informative is the consideration of the Raman spectroscopy and the EPR not as simply correlated but rather as complementary methods focused on highlighting features best revealed by each method.

Among the most actively developed applications of such biomedical molecular research is the activity of antioxidant substances in skin tissues. In the work [187], the magnitude decay of EPR peaks with time allowed the specification of the kinetics representing the nitroxide reduction due to antioxidants and the determination of the respective rate constant. In turn, resonance Raman spectroscopy, which detects the target antioxidant species, carotenoids, was used to correlate the concentration of the latter to these EPR-based rates.

The chemical structure of carotenoids and their radical-related reaction allows one to quite clearly access the involved unpaired electrons by EPR spectroscopy, which ensures this method is an accurate and informative approach for both EPR-based investigations [85,86] and Raman spectroscopy based ones. The usage of resonance Raman spectroscopy for the noninvasive testing of beta-carotene and related substances was pioneered in the work [188].

The recent work [189] utilised EPR measurements to quantify the radical formation in vivo during the skin treatment by herbal oils as a time function, while the Raman spectroscopy gave information on the penetration depth using specific herbal compounds’ spectral fingerprints. Therefore, this combined spatiotemporal information may play a role in the background for the future building of a kind of reaction–diffusion model of the process. Some studies [190,191] combining both approaches were used to explore the drought and oxidative stresses in plants. The resonance Raman microspectroscopy was used to detect carotenoids, which are involved in molecular interactions related to driving photosynthesis and protecting plants from overexposition to sunlight (in leaves) and the spatial distribution of proteins, fats, and starch (in grains). Simultaneously, EPR spectroscopy allowed the determination of the number of radical species in whole grains and the distinction between stress-tolerant and stress-sensitive wheat cultivars, as well as the characterisation of the protective mechanisms against physical stress in plants’ leaves. A number of works argue in favour of combining EPR spectroscopy, which allows the measurement of short-lived radicals existing in low concentrations and resulting in oxidative stress, and resonance Raman spectroscopy for the simultaneous determination of redox-related biomarkers. In particular, the interdependence between oxidative and antioxidant processes in secondary keratinocytes treated with various beta-carotene concentrations was studied with such a combination of experimental methods in the work [192]. More recently, such a scheme was applied [36] to the case of blood plasma affected by oxidative stress when beta-carotene is considered an antioxidant.

Other types of biomolecules, to which combined methods are applicable, include metal–organic compounds especially involved in the oxygen-carrying and reduction processes. Despite the fact that realising an idea of the complex characterisation of heme-related complexes was initiated several decades ago, see e.g., [193], its deep development, which takes advantage of both methods, has started to be developed relatively recently. The work [194] addressed the complex multimodal exploration of chlorite dismutase-like (Cld-like) proteins, which are known as HemQs (coproheme decarboxylase) in Gram-positive bacteria, based on the complementarity of methods revealing structural and metabolic functions. The simultaneous usage of UV–visible absorption and EPR and resonance Raman spectra allowed the obtention of comprehensive spectroscopic signatures of ferric wild-type NdCld as well as some mutant variants. The key idea of such a complex characterisation is in the coordination of high-spin states registered with the EPR and the bond-based vibrational bands registered by the RR. This line of complementary characterisation of heme-related biomolecules was generalized in the case of protein-ligand binding, e.g., [195,196], which led to the modern view of $H_{2}O_{2}$-mediated oxidation reactions as related to the detailed structure of the involved molecules [197,198,199]. Similar approaches combining the EPR-based information at the active site’s structure with the data obtained with the RR found their applications to other kinds of hydrogenases as well [200,201]. Moreover, they provide recent perspectives for the synthesis of artificial analogues to related biomimetic molecular complexes [202,203]. Among modelling approaches aimed at a better understanding of oxidative stress mechanisms and the specificity of different substances, machine learning starts to drastically gain growing attention [204]. Among recent results which address the usage of both Raman and EPR-based characteristics as the input data for machine learning algorithms aimed at the accelerated discovery of nanozymes, one can mention the work [205], where a family of metalloenzymes catalysing the dismutation of superoxide anion radical into biologically less harmful species was considered. In this case, Raman spectroscopy was used to determine the symmetry group of the active molecules (transition metal triphosphates $M_{x}S_{y}P_{z}$), and the acceptor capacity of these compounds to $O_{2}^{-}$ was obtained by EPR spectroscopy. Meanwhile, various machine learning algorithms, including logistic regression, support vector machine, decision tree with gradient boosting, multilayer perceptron, extreme gradient boosting (XGBoost), convolutional neural network, light gradient boosting machine, adaptive boosting, and random forest were used to optimise $M_{x}S_{y}P_{z}$ aimed at achieving the desired biochemical outcome.

### 5.2. Combining EPR and DSC Methods

The review [87] summarises earlier works on the features revealed for antioxidant compounds by the EPR methods; among them, one can mention the following: polar carotenoids can either increase or descend the membrane fluidity depending on their phase state, ordered phase or liquid crystalline, respectively. Recently, this interpretation of the EPR-based data received a detailed confirmation of the microscopic mechanisms by all-atom MD simulations [88]. At the same time, another experimental approach, which directly addresses this feature, is the differential scanning calorimetry. In the recent work by Chia et al. [206], this was illustrated by the case study of mitigating *Escherichia coli*’s membrane fluidization by the presence of two polar carotenoids, lutein and zeaxanthin. The discovered effect is of special interest from the point of view of biofuel production by bacterial cultures since it improves the tolerance of bacterial cells to the produced biobutanol.

As a very illustrative example of the direct correspondence between the DSC and EPR data, one can consider the results reported by J. Velasco et al. [180] who studied the oxidative stability of vegetable oils. For this goal, the tendency to radical formation was monitored. The key parameter determined in this work was the induction period, which indicates the time required for the transition from slow oxidation to the onset of a sudden increase in oxidation rate. Measurements were carried out by isothermal DSC at $100{\;}^{\circ }C$ and by the EPR method at $60{\;}^{\circ }C$ for a variety of 11 different vegetable oil samples. They included rapeseed oil and sunflower oil, their mutual mixtures and mixtures with the addition of α-tocopherol (a type of vitamin E involved in the process of lipid homeostasis under oxidative conditions). Figure 6B demonstrates the interrelation of the induction periods obtained by the two methods in two rounds of equivalent experiments. One can see that although the period’s numerical values are not equal, they are very well linearly correlated (correlation coefficients are equal to 98% and 97% for the first (squares) and the second (diamonds) experiment, respectively). Thus, one can choose more accessible equipment for investigations in this field. Enzymatic control of such processes can also be addressed using both EPR and DSC methods. In particular, the correlation of the signals obtained by these two methods was noted in the study of the bovine liver catalase described in the work [207].

Another area of active recent interest in this field is the formation and functioning of liposomes, which follows the path proposed by L. Zhao et al. [174]. In the cited work, a shift of the phase transition temperature was registered by DSC (melting depending on fluidity) for different concentrations of paclitaxel, one antineoplastic agent, included in the phospholipids, cholesterol, and dipalmitoylphosphatidylcholine-based liposomes. Simultaneously, an EPR signal’s shift under the same concentration conditions was registered, which allowed for a more detailed microscopic interpretation of the effect of paclitaxel’s binding. The work [208] reports tuning the properties of liposomes formed by 25 different lipids belonging to the phosphatidylcholine, phosphatidylethanolamine, phosphatidylglycerol, and phosphatidylserine types. In that case, the separation between the spectral extrema of the EPR signal reflects the rotational motional freedom near the polar head regions of the bilayer, indicating an increase in its fluidity. Simultaneously, the DSC signal reflects the temperature-dependent passage from the rippled gel phase to the liquid crystalline phase. Choosing and adjusting the fluidity of liposomes is crucial for their usage as containers for drug delivery since this property defines the interaction with the biological systems at the cell level, e.g., with the mucus layer for pulmonary drug delivery, skin barriers for transdermal delivery, as well as the drug release at the target place of an organism. Combining the DSC measurements, which characterise the stability of liposomes, with the EPR data, reflecting an interaction of the liposome encapsulating a drug with the latter, was explored in the work [209] for besifloxacin liposomes intended for ocular drug delivery. The work by Pantak et al. [210] combines these two methods to reveal the effect of the surrounding medium’s pH value on the properties of drug-encapsulated liposomes within a similar protocol of study to when the EPR-based signal is in charge of the behaviour of a drag-mimicking marker and the DSC measurements report the properties of the envelope. Note also that liposomes can serve not only as containers for drugs interacting with an organism’s cells but also as physicochemical models of a cell membrane itself. A separate review of the respective works, which also includes combinations of the DSC and EPR methods, is given by the paper [25].

## 6. Conclusions

Thus, the main message of this review can be formulated as follows. The considered experimental techniques (EPR, Raman spectroscopy, and DSC) require pieces of equipment which are relatively widespread in physical laboratories as they provide tools for a range of investigations. Biophysical studies, which address molecular complexes and interactions in macroscopic organisms, can be placed in their row. Each of these approaches has its own specificity, which allows the obtention of a more comprehensive picture of underlying processes. Due to their “macroscopic” character, both EPR and DSC methods give an averaged picture of the state change process of the overall sample. The DSC method addresses, first of all, the simple revealing phase changes in the explored systems, either induced by experimental temperature growth, mimicking the change in the system’s energy or accompanying another reaction process (in the isothermal mode of measurements). Such changes may originate from their restructuring, e.g., due to a change in the fluidity of cell membranes or biochemical reactions alternating molecular complexes. This method is the simplest in physical experimental implementation but is the least selective. The EPR method also provides similar averaged information, but it is more selective since changes in the magnitude and the shift of the signal’s components can be correlated with particular molecular compounds involved in biochemical reactions. In short, EPR spectroscopy can be used to obtain specific information on the progress, outcome, and kinetic constants of a particular process of interest. At the same time, Raman spectroscopy is not only more sensitive to details of intramolecular oscillations accompanying such reactions as well as of chemical constituents of biological objects (e.g., cell wall in general, proteins included in its content, etc.) but can also be focused more accurately at particular points of interest, giving a more detailed spatial mapping. A combination of pairwise or triple combinations of three approaches can provide a complimentary picture. Thus, three considered physical methods, which gained recent attention in biomolecular biophysical and biomedical studies, actually open new wide perspectives and deserve a wider development in this field of study.

## Data Availability

The present work is a review; any data request should be addressed to the authors of original works.
